# Identification of an *evx1*-Dependent Joint-Formation Pathway during FIN Regeneration

**DOI:** 10.1371/journal.pone.0081240

**Published:** 2013-11-20

**Authors:** Quynh V. Ton, M. Kathryn Iovine

**Affiliations:** Department of Biological Sciences, Lehigh University, Bethlehem, Pennsylvania, United States of America; University of Sheffield, United Kingdom

## Abstract

Joints are essential for skeletal flexibly and form, yet the process underlying joint morphogenesis is poorly understood. Zebrafish caudal fins are comprised of numerous segmented bony fin rays, where growth occurs by the sequential addition of new segments and new joints. Here, we evaluate joint gene expression during fin regeneration. First, we identify three genes that influence joint formation, *evx1*, *dlx5a*, and *mmp9*. We place these genes in a common molecular pathway by evaluating both their expression patterns along the distal-proximal axis (i.e. where the youngest tissue is always the most distal), and by evaluating changes in gene expression following gene knockdown. Prior studies from our lab indicate that the gap junction protein Cx43 suppresses joint formation. Remarkably, changes in Cx43 activity alter the expression of joint markers. For example, the reduced levels of *Cx43* in the *sof* ^*b123*^ mutant causes short fin ray segments/premature joints. We also find that the expression of *evx1-dlx5a-mmp9* is shifted distally in *sof* ^*b123*^, consistent with premature expression of these genes. In contrast, increased *Cx43* in the *alf* ^*dty86*^ mutant leads to stochastic joint failure and stochastic loss of *evx1* expression. Indeed, reducing the level of *Cx43* in *alf* ^*dty86*^ rescues both the *evx1* expression and joint formation. These results suggest that *Cx43* influences the pattern of joint formation by influencing the timing of *evx1* expression.

## Introduction

The precise location of joints provides both flexibility and form to the vertebrate skeleton. We use the zebrafish regenerating fin as a model to study skeletogenesis, including the appropriate formation of fin ray joints. Fin ray joints have been termed “fibrous joints” [1] since the articulation occurs in the bone matrix while the central mesenchyme remains continuous. These joints are distinct from synovial joints which completely articulate previously uninterrupted cartilaginous templates of the endochondral skeleton [2]. The fin grows rapidly during regeneration, fully restoring fin size and pattern. The fin is comprised of multiple bony fin rays or lepidotrichia, and each fin ray is comprised of multiple bony segments separated by fin ray joints (or simply, joints). Each fin ray consists of two hemirays of bone matrix surrounding the mesenchyme, and several layers of epidermal cells surrounding the bone matrix. Actinotrichia are bundles of collagen-like fibers that emanate from the distally located basal epidermal cells and serve as a substrate for osteoblasts to align and secrete bone matrix directly [3]. The mesenchyme medial to the actinotricha includes dividing cells, undifferentiated cells, blood vessels, nerves, and connective tissues [4,5]. The mesenchyme lateral to the actinotrichia includes the bone matrix, osteoblasts, and joint-forming cells [6,7]. Joint-forming cells are a subpopulation of lateral mesenchymal cells that condense on the surface of the uninterrupted bone matrix and form an elongated row of cells at the site of the presumptive joint. These cells later separate into two rows of cuboidal cells that flank a newly established articulation in the bone matrix [7]. Thus, these cells appear responsible for the articulation of the fin ray jointsWe refer to the osteoblasts and joint-forming cells collectively as skeletal precursor cells.

During growth and regeneration, the fin regenerates in the proximal to distal direction where new segments and new joints are continually added to the distal end of the fin ray. Thus, youngest tissue is always located more distally than mature tissue [8]. Following amputation, the regenerate undergoes three main stages: wound healing, blastema formation, and outgrowth [5,9]. The blastema is a specialized compartment comprised of rapidly proliferating cells, and is located in the distal and medial mesenchyme. These cells are the source of new tissue during regeneration. Recent studies show that several cell types in the regenerating fin are lineage restricted, meaning that new cells in the regenerating fin arise from precursor cells of the same cell type [10-13]. These cells undergo de-differentiation, cell proliferation, and re-differentiation, in order to replace lost tissue. This may not represent the only way to replace lost tissue, as others have found that osteoblasts are capable of de novo differentiation during fin regeneration [11]. Osteoblasts and joint-forming cells appear to be derived from a common lineage [13]. To date, little is known about the genes required for differentiation of joint-forming cells, or indeed, the signals required to initiate this process.

The transcription factor Even-skipped 1 (Evx1) belongs to a family of vertebrate eve-related homeobox genes [14]. In zebrafish regenerating fins, the expression of *evx1* was observed strongly in the distal-most and youngest joints [1]. Sections of *evx1* following *in situ* hybridization (ISH) showed a strong expression level of *evx1* mRNA in the lateral compartment where skeletal precursor cells reside [1]. More recently, *evx1* was shown to be required for joint formation since an *evx1* mutant fails to produce fin ray joints during regeneration [15]. Our evaluation of two other fin mutants, *short fin* (*sof* ^*b123*^) and *another long fin* (*alf* ^*dty86*^), suggest that the gap junction protein Connexin43 (Cx43) also contributes to joint formation. Both *cx43* mRNA and Cx43 protein are expressed throughout the medial mesenchyme, adjacent to the lateral populations of skeletal precursor cells [16]. The *sof* ^*b123*^ mutant exhibits reduced levels of *cx43* mRNA and protein (without a lesion in the coding sequence) that lead to reduced cell proliferation, short segments (i.e. premature joints) and short fin length [17]. In contrast, the *alf* ^*dty86*^ mutant exhibits fin overgrowth and overlong segments on average due to stochastic joint failure [18]. The *alf* ^*dty86*^ phenotype is not caused by mutations in *cx43* but coincidently has increased levels of *cx43* mRNA [7]. We have shown that morpholino-mediated *cx43* knockdown in *alf* ^*dty86*^ rescues joint formation, suggesting that the higher levels of *cx43* in this mutant contributes to the loss of fin ray joints [7]. Thus, reduced *cx43* leads to premature joints while increased *cx43* leads to joint failure. We interpret these findings to indicate that Cx43 suppresses joint formation, perhaps by communication between the medial *cx43*-positive mesenchyme and the lateral *evx1*-positive mesenchyme.

As an initial attempt to understand the events initiating and controlling joint formation, we first wished to define additional molecular players acting downstream of *evx1*. Here, we describe the addition of two *evx1*-dependent joint gene markers that also contribute to joint formation: *distal-less homeobox-5a* (*dlx5a*) and *matrix-metalloproteinase-9* (*mmp9*). We also exploited the characteristics of low and high Cx43 activity in *sof* ^*b123*^ and *alf* ^*dty86*^ to address the relationship between the expression of these joint genes and Cx43 activity during joint patterning. We found that the onset of joint gene expression correlates with the level Cx43 activity. These results suggest that Cx43 may regulate joint formation by influencing the timing of *evx1* expression.

## Materials and Methods

### Statement on the ethical treatment of animals

This study was carried out in strict accordance with the recommendations in the Guide for the Care and Use of Laboratory Animals of the National Institutes of Health. The protocols used for this manuscript were approved by Lehigh’s Institutional Animal Care and Use Committee (IACUC) (protocol identification 128, approved 11/14/2012). Lehigh University’s Animal Welfare Assurance Number is A-3877-01. All experiments were performed to minimize pain and discomfort.

### Fish maintenance

Zebrafish were derived from the C32 strain. Mutant fish used in these studies include *sof* ^*b123*^ [19], *alf* ^*dyt86*^ [18], homozygous evx1^-/-^ mutant fish, and heterozygous carriers [15]. All fish were raised and cared for at constant temperature of 25°C in a 14 light: 10 dark photoperiod [20].

### RNA probes and whole mount in situ hybridization (ISH)

Antisense *evx1* probe was generated from 1µg of a PCR-generated linear template containing a T3 RNA polymerase binding site (F-TAATACGACTCACTATAG; 

R-T3- GGATCCATTAACCCTCACTAAAGGGAAGAGCTATGACGTCGCAT where the T3 binding site is underlined). Antisense digoxigenin-labeled *shh*, *lef1*, *mmp9*, and *dlx5a* probes were generated from *lef1* cDNA [21], *shh* cDNA [21], *mmp9* cDNA [22], and *dlx5a* cDNA [22]. The template for the *col10a1b*probe was a generous gift from the lab of Dr. David Parichy (representing sequence with genbank ID # 68437010).

Whole-mount ISH was performed on 5 dpa regenerating fins as described [23]. Stained fins were examined on a Nikon Eclipse 80i microscope. Images were collected using a digital Nikon camera. At least 4 regenerating fins were assessed at a time, and all markers were examined in triplicate.

### ISH on cryo-sections

Fin regenerates (5 dpa) were harvested and fixed overnight with 4% paraformaldehyde in PBS. Fins were dehydrated in 100% methanol at -20°C. Next, fins were rehydrated in a methanol-PBS series of washes before embedding in 1.5% agarose/5% sucrose and equilibrated overnight in 30% sucrose. Blocks were mounted in OCT and cryosectioned (15 µm sections) using a Reichert–Jung 2800 Frigocut cryostat. Sections were collected on Superfrost Plus slides (Fisher) and allowed air dry overnight at room temperature. Slides may be stored in a freezer box at -20°C for up to one year. A marking pen (ImmEdge™ Pen H-4000; PAP pen, Vector Laboratories) was used to circle the sections. Probe was pre-hybridized with a mixture of 1X salt solution (NaCl, Tris HCl, Tris Base, Na_2_HPO_4_.7H_2_0, NaH_2_PO_4_, and 0.5 M EDTA) with 50% deionized formamide (Sigma), 10% dextran sulfate, 1mg/mL tRNA, and 1X Denhart’s (Fisher) at 70°C for 5 mins. Hybridization with digoxigenin-labeled antisense probes was completed overnight at 65°C. The next day, slides underwent series of washes in a solution that has 1X SSC, 50% formamide and 0.1% Tween-20 at 65°C. Slides were then transferred to room temperature for extensive washes in MABT (100 mM Maleic acid, 150 mM NaCl, and 0.1% Tween-20) before incubation in blocking solution (MABT, Goat serum and 10% milk) for at least 2 hours or overnight. Anti-digoxigenin Fab fragments (pre-absorbed against zebrafish tissue) were used at 1:5000 overnight at 4°C. After incubation, slides were washed in MABT four times followed by two short washes in staining buffer (100mM Tris, 9.5, 50 mM MgCl_2_, 100mM NaCl, and 0.1% Tween20). Slides were next transferred to 10% polyvinyl alcohol (PVA; MW: 86,000) staining solution plus NBT/BCIP stock solution (Roche) and development proceeded overnight at 37°C. Once observing purple staining on the sections, the reaction was stopped by washing the slides with PBST for at least 3 hours. Sections were mounted in 100% glycerol and examined on a Nikon Eclipse 80i microscope. Images were collected using a digital Nikon camera. A minimum of 10 sections were evaluated for each of a minimum of two different fins.

### Morpholino-mediated gene knockdown and electroporation

Injection and electroporation experiments were performed as described previously [7,16,23,24]. Targeting morpholinos were designed against the start codon and modified with fluorescein at the 3’ end (Gene Tools, LLC) to provide a charge and for detection. Control morpholinos were either custom mismatch morpholinos containing five mismatches to the targeted gene or were the Gene Tools ‘standard control’ morpholino, which does not recognize any zebrafish genes. Following injection and electroporation, fins were harvested at 1day post electroporation (dpe) to evaluate changes in gene expression. At least 4 regenerating fins were treated per morpholino (targeting or control), and all knockdown experiments were completed in triplicate.Morpholino sequences for *cx43* were described previously [16]. Morpholinos used here include: *evx1*-MO, CTTTCCGTGCTTCGGCGAGCCCATT; *evx1*-MM, CTTTGCCTGGTTCGGCCACCCCATT; *mmp9*-MO, AAACGCCAGGACTCCAAGTCTCATC; *dlx5a*-MO, CGAATACTCCAGTCATAGTTTGGAT (also used in [25]); Standard control MO, CCTCTTACCTCAGTTACAATTTTATA. 

### Measurements

Measurements of the distal boundaries of ISH expression domains to the distal end of the fin were taken from the third fin ray (V+3) since that was established as a standard [19]. Student’s t-tests were performed to determine if data sets were statistically different (p < 0.05). At least 8 fin rays per marker (*evx1*, *dlx5a*, *mmp9*, *col10a1b*, *shh*, and *lef1*) were measured.

## Results and Discussion

### *dlx5a* and *mmp9* are expressed downstream of *evx1*

**Figure 1:**
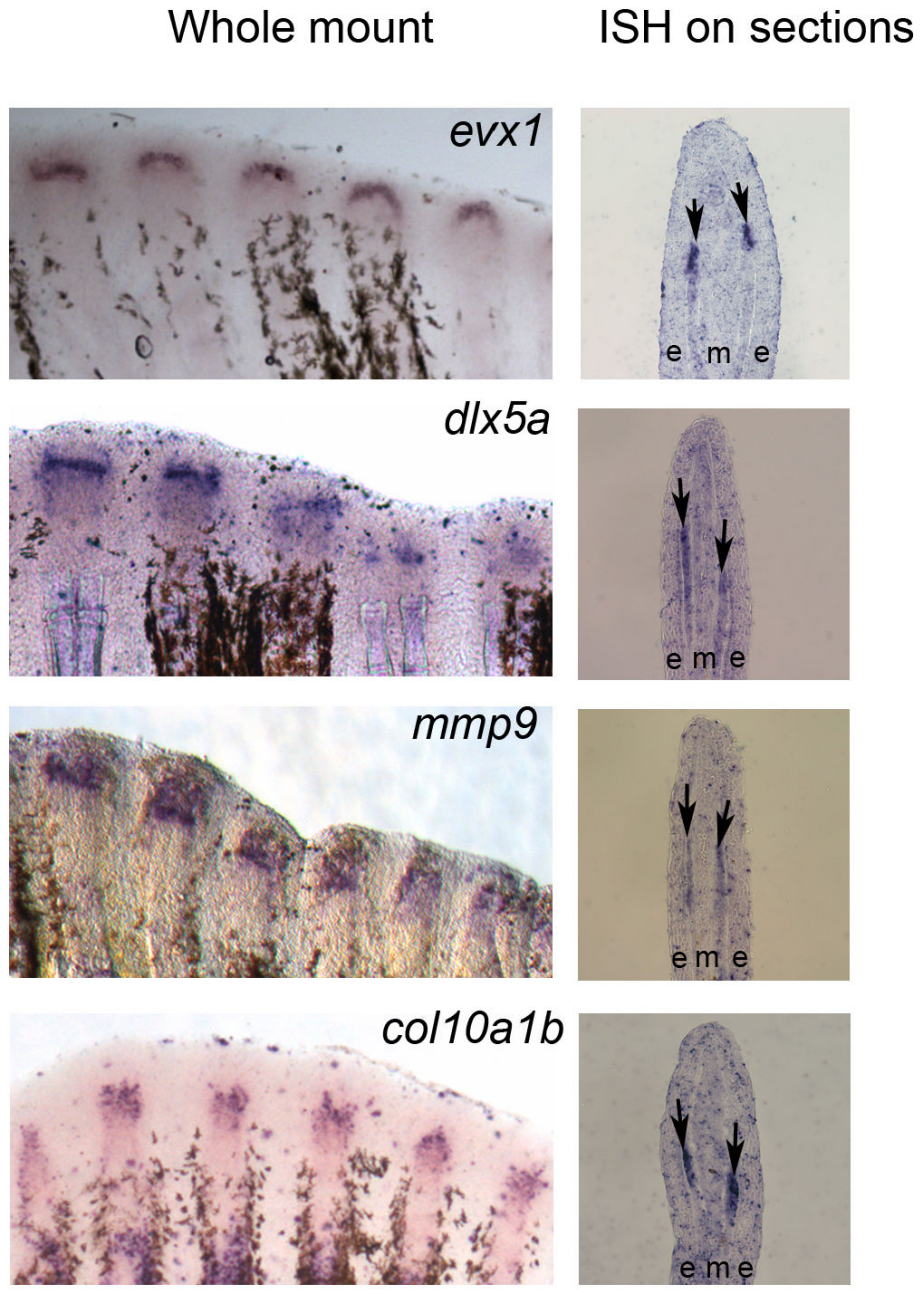
Expression of joint genes in regenerating fins. (Left) Whole mount ISH shows *evx1*, *dlx5a*, *mmp9*, and *col10a1b* are expressed in 5 dpa wild type fins. (Right) ISH on wild-type 5 dpa cryosections reveal expression of joint genes in the lateral skeletal precursor cells. Arrows point to gene expression in the skeletal precursor compartment. (e) epithelium; (m) mesenchyme.

Since our studies suggest that Cx43 influences joint formation, we were interested in identifying additional genes that function together to regulate this process. Unlike osteoblast genes which are expressed in broad domains throughout the lateral compartment in the regenerating fin [8], genes expressed during joint formation tend to be expressed in a discrete group of cells. The expression pattern typically appears as a band of cells following whole mount in situ hybridization (ISH), and these cells are located within the lateral population of skeletal precursor cells (i.e. see 1). Thus, we identified *evx1*, *dlx5a*, *mmp9*, and *col10a1b* as candidate joint genes based on their location of expression [1,22]. We first confirmed the location of gene expression of this set of genes using whole mount ISH on 5 days-post-amputation (dpa) regenerating fins and by ISH on cryo-sectioned tissue of 5 dpa caudal fins. As expected, we found *evx1*, *dlx5a*, *mmp9* and *col10a1b* are strongly expressed in a discrete group of cells in the lateral compartment where the skeletal precursor cells reside (Figure 1).

**Figure 2:**
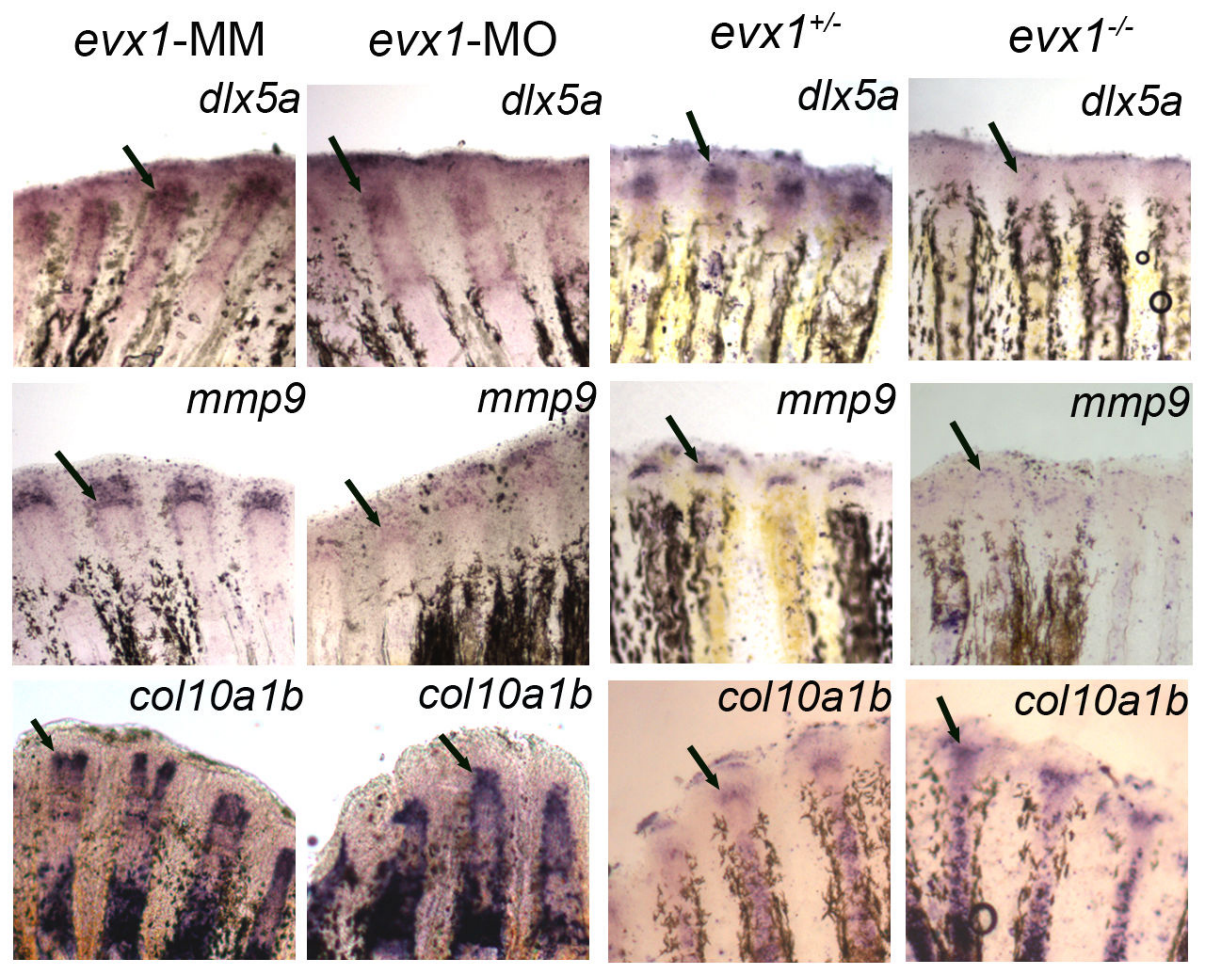
*dlx5a* and *mmp9* are genes downstream of *evx1*. (A) Whole mount ISH shows levels of *dlx5a* expression and *mmp9* expression are reduced in the *evx1*-morpholino (*evx1*-MO) injected side compared with the *evx1*-mismatch (*evx1*-MM) injected side, while the level of *col10a1b* expression is unchanged. (B) Whole mount ISH on *evx1* ^*-/-*^ mutants displays similar results seen in the *evx1*-MO injected fins, except that a stronger reduction in *dlx5a* and *mmp9* is observed. Arrows identify regions of the fin where staining is present and/or expected (i.e. in the cases where reduced *evx1* influences expression levels).

**Figure 3:**
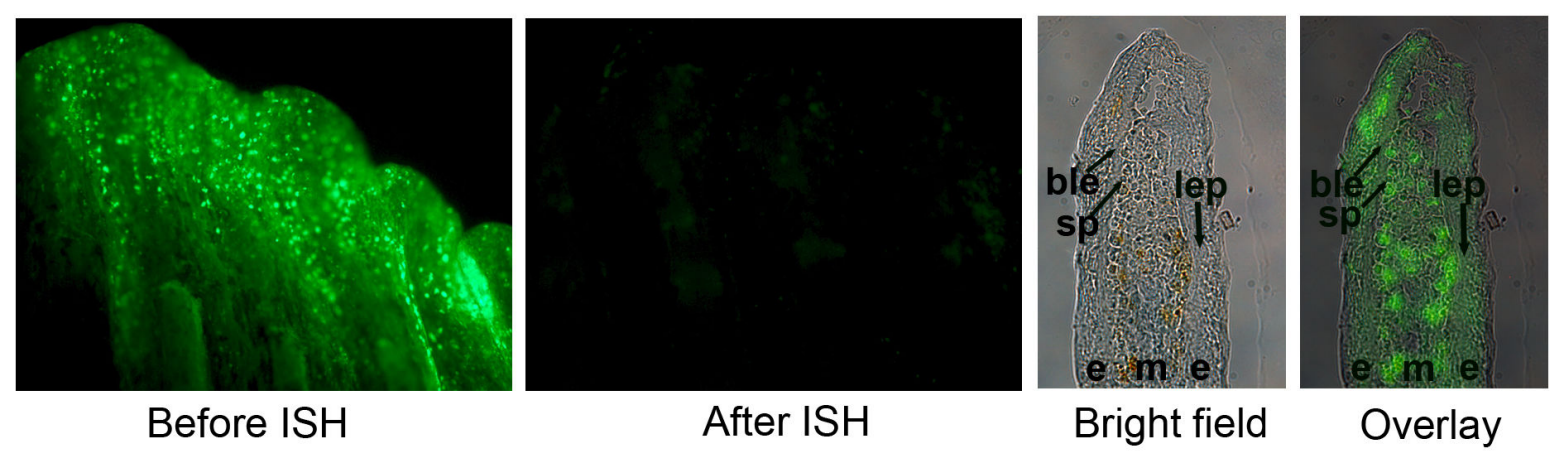
Morpholinos target all cellular compartments of the regenerating fin. Fluorescein-positive cells successfully took up the morpholino (1 day post electroporation). The two left panels reveal loss of fluorescein signal following in situ hybridization. The two right panels demonstrate that fluorescein-positive cells are observed in all cellular compartments in freshly harvested and cryosectioned fins. The basal layer of the epidermis (ble) is identified as a row of cuboidal cells between the epithelium (e) and the mesenchyme (m). The skeletal precursor cells (sp) are located adjacent to the ble. Morpholino uptake is observed in the outer epithelial layers, in the skeletal precursors, and in the medial mesenchyme.

It has been proposed that *evx1* is one of the earliest joint gene markers [1]. Indeed, evx1^-/-^ mutants lack fin ray joints, demonstrating that *evx1* is required for joint formation [15]. We have also found that morpholino-mediated knockdown of *evx1* is sufficient to cause joint failure (data not shown). We next investigated if expression of *dlx5a*, *mmp9*, and *col10a1b* depend upon *evx1* for their expression by taking advantage of both morpholino-mediated knockdown of *evx1* and the evx1^-/-^ mutant fins. We expected to find that expression of *evx1*-dependent genes is reduced in the knockdown fins and completely absent in the evx1^-/-^ mutant fins. Indeed, we found that expression signals of *dlx5a* and *mmp9* are reduced in *evx1*-knockdown fins, while *col10a1b* expression appeared unaffected (Figure 2). One possibility for failure to observe a knockdown effect on *col10a1b* expression is that the *evx1*-morpholino did not target the *col10a1b*-expressing cells located in the lateral mesenchyme. However, it was not possible to evaluate cells doubly-labeled for the fluorescein-tagged morpholino and for gene expression since the fluorescein signal is labile following in situ hybridization (Figure 3). Examination of the location of fluorescein signal prior to in situ hybridization reveals that the *evx1*-morpholino targets all compartments of the regenerating fin, including the lateral compartment of skeletal precursor cells (Figure 3). Together with the finding that the *evx1*-morpholino targets the *dlx5a* and *mmp9*-expressing cells located in the same compartment, these findings strongly suggest that morpholinos regularly target the lateral mesenchymal cells during gene-knockdown. We next evaluated gene expression in evx1^-/-^ regenerating fins. Similar to our findings using the *evx1*-morpholino, we find that expression of *dlx5a* and *mmp9* are more severely reduced in *evx1* ^*-/-*^ regenerating fins, while *col10a1b* is also not affected in those fins (Figure 2). Interestingly, *dlx5a* and *mmp9* are not completely abolished in the *evx1* ^*-/-*^ mutants, suggesting that an alternate, non-*evx1*-dependent pathway may also contribute to expression of these genes. Taken together, these data suggest that *dlx5a* and *mmp9* are expressed downstream of *evx1*, while *col10a1b* is not. Continued studies therefore focused on *dlx5a* and *mmp9*. Both *evx1* and *dlx5a* encode for homeobox domain-containing transcription factors, although their direct targets are largely unknown. The *mmp9* gene codes for a matrix metalloprotease enzyme, which is responsible for degradation of extracellular matrix proteins. During the process of joint morphogenesis, the previously uninterrupted bone matrix separates into two bony elements [7]. It is possible that Mmp9 activity contributes to this articulation event through digestion of the bone matrix.

**Figure 4:**
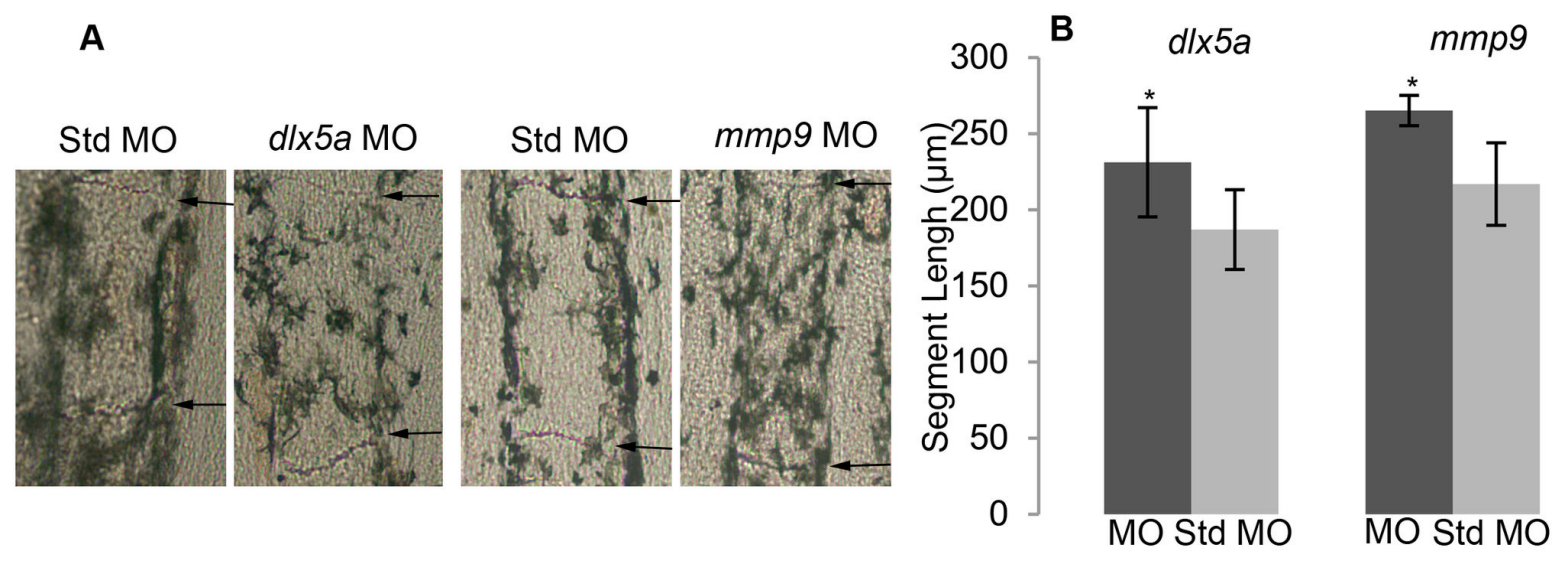
*dlx5a* and *mmp9* are necessary for correct joint placement. (A) Segment length is increased following targeted gene knockdown of *dlx5a* and *mmp9* compared with standard control (std) morpholino knockdown (negative control). (B) Segment length is increased in *dlx5a*-knockdown and *mmp9*-knockdown fins compared with standard control morpholino. Statistically different data sets (*) were determined by the student’s t-test where p<0.05. The p-value for the comparison of segment length for the *dlx5a*-treated fins was p = 0.0047. The p-value for the comparison of segment length for the *mmp9*-treated fins was p = 0.0018. Error bars represent the standard deviation. MO, morpholino.

We next wished to distinguish between the *dlx5a* and *mmp9* genes acting simply as joint markers or as providing a function during joint formation. Reduced function of genes required for joint formation is predicted to cause either complete joint failure or a delay of joint formation (i.e. longer segments). We find that morpholino-mediated knockdown of *dlx5a* and *mmp9* both cause increased segment length (Figure 4). Importantly, the knockdown of these genes represents the first example of a manipulation causing longer segments in the fin. Indeed, the only other example of long fin ray segments is the *alf* ^*dty86*^ mutant. We cannot rule out the possibility that *dlx5a* and *mmp9* may also influence the rate of fin growth. However, changes in growth rate are not sufficient to influence segment length. Fish grown in crowded conditions grow slower than fish grown in sparse conditions, but segment length is not different between these groups [19]. Moreover, abrogation of either Fgfr1 or Shh, while influencing the rate of cell proliferation and fin length, do not influence segment length [26,27].Thus, our findings support a model where *dlx5a* and *mmp9* contribute to correct joint placement, irrespective of any putative role in regulating fin growth. 

### Placing the genes of the joint pathway in a linear order

**Figure 5:**
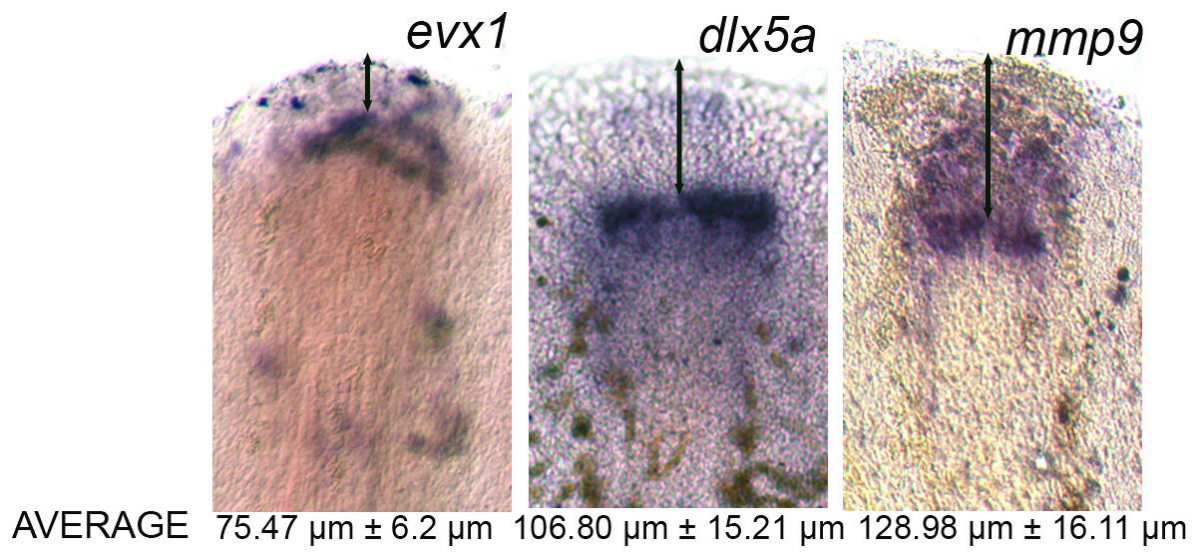
Expression domains of joint genes expressed during fin regeneration. The double arrow identifies the measured distance between the expression domains and the distal tip of the fin. The measurement was taken from the third ray of each fin and the average and standard deviation (±) were calculated.

Previously, our lab showed that the early genes required for osteoblast differentiation initiated in the more distal, less mature osteoblasts, while onset of expression of late osteoblast genes was observed in the more proximal, more mature osteoblasts [8]. Here we applied a similar approach for this set of joint-forming genes in an attempt to reveal a preliminary order of the *evx1*-dependent genes. We completed whole mount ISH at 5 dpa and measured the distance of expression domains of *evx1*, *dlx5a*, and *mmp9* to the distal end of the fin. As anticipated, *evx1* is expressed in the most distal domain of skeletal precursor cells, consistent with this gene acting the earliest (Figure 5). Since *dlx5a* and *mmp9* appear downstream of *evx1*, we expected to find their gene expression more proximally. We find that *dlx5a* is expressed more proximally than *evx1*, while *mmp9* is expressed more proximally than both *evx1* and *dlx5a* (Figure 5). These findings support the hypothesis that *dlx5a* and *mmp9* are expressed downstream of *evx1* and further suggests the following linear pathway: *evx1* followed by *dlx5a* followed by *mmp9*. 

**Figure 6:**
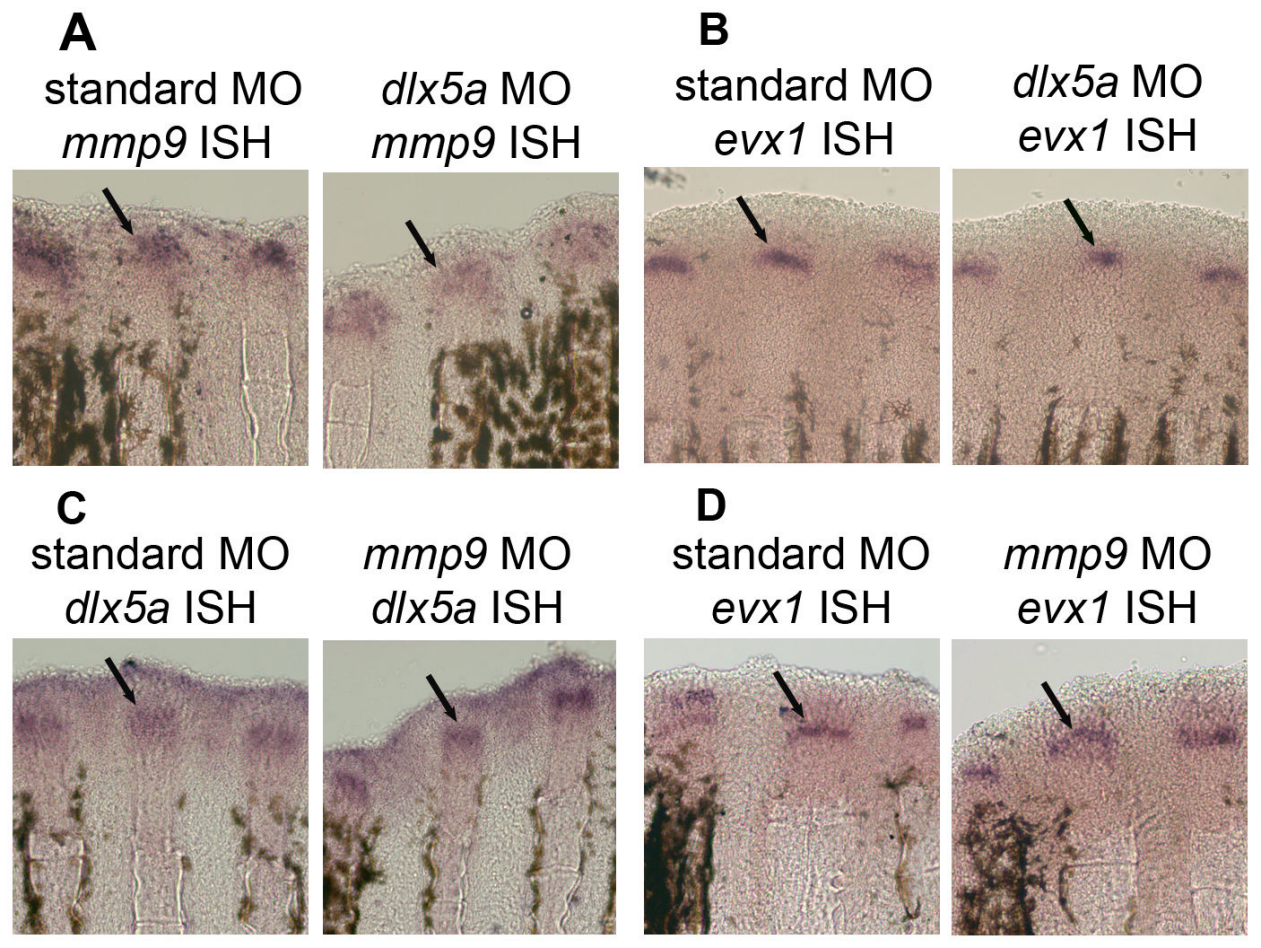
Confirmation of the predicted *evx1*-dependent joint pathway. Morpholino-mediated gene knockdown followed by whole mount ISH show that *dlx5a*-knockdown causes reduced *mmp9* expression (A) but does not influence *evx1* expression (B). *mmp9*-knockdown does not influence *dlx5a* expression (C) or *evx1* expression (D). Arrows point to the in situ hybridization staining.

To confirm this predicted order of gene expression, we examined changes in gene expression following *dlx5a* **-**knockdown and *mmp9* **-**knockdown by whole-mount ISH (Figure 6). We found that *mmp9* expression is reduced in fins treated for *dlx5a*-knockdown, consistent with the hypothesis that *mmp9* is expressed downstream of *dlx5a*. In contrast, *dlx5a* expression is not affected by *mmp9* **-**knockdown. Similarly, *evx1* expression is not affected by either *dlx5a*-knockdown or by *mmp9* **-**knockdown. Together with our earlier findings that loss of *evx1* causes reduced expression of *dlx5a* and *mmp9* (i.e. Figure 2), these results confirm the relative order of gene expression predicted by the expression patterns along the proximal-distal axis and further suggests that *dlx5a* and *mmp9* function does not feedback on expression of *evx1*. 

### Cx43 regulates the evx1-dependent joint pathway

**Figure 7:**
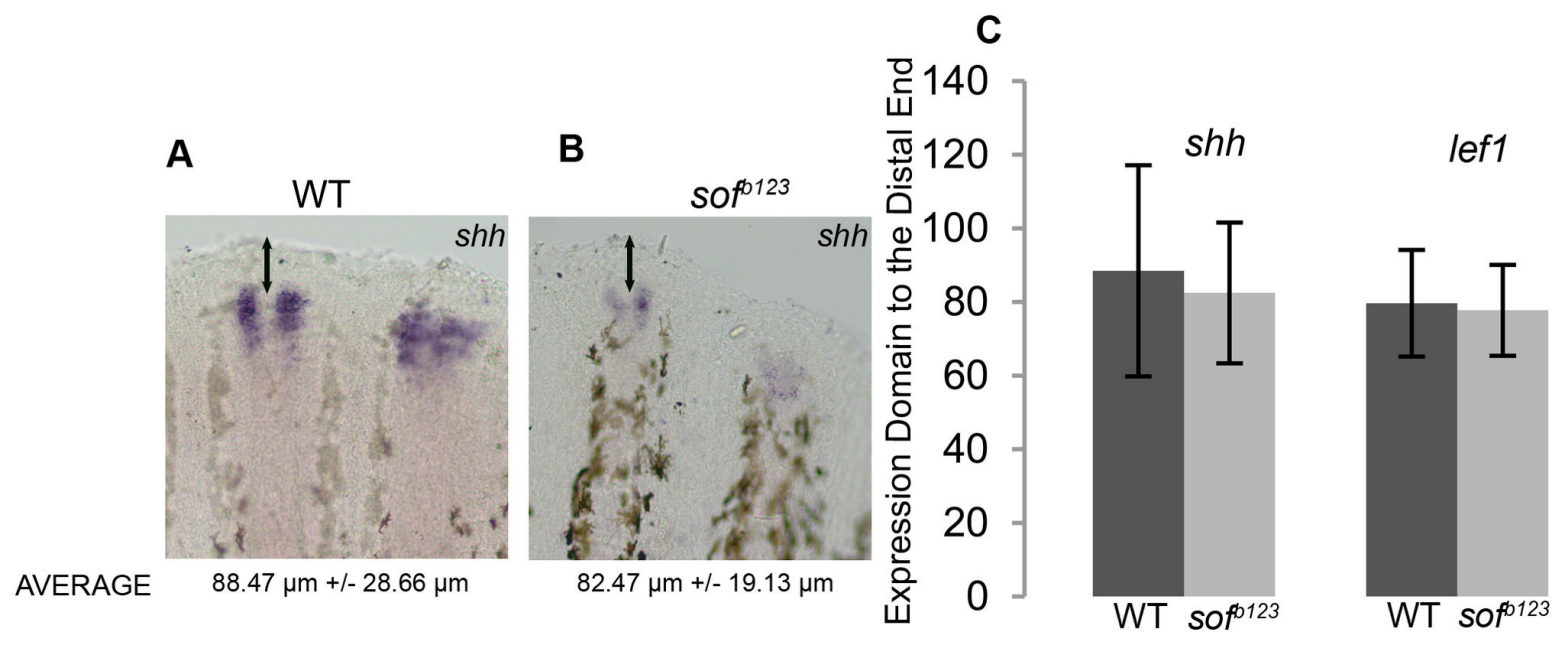
Reduced growth rate of *sof* ^*b123*^ mutants does not influence patterning of gene expression. (A) Whole mount ISH of *shh* on WT fins (A) and on *sof* ^*b123*^ fins (B). (C) The distance of expression of *shh* and *lef1* to the distal end of the fin is not influenced by the reduced growth rate of *sof* ^*b123*^ since the distances of the expression domains for *shh* and *lef1* are not statistically different by the student’s t-test (p>0.05). The p-value for comparison of the *shh* domain was p = 0.19. The p-value for the comparison of the *lef1* domain was p = 0.30. Arrows identify the region of the fin that was measured, from the distal expression domain to the distal end of the fin. Error bars represent the standard deviation.

Based on our previous findings, we have suggested that Cx43 activity in the medial compartment, adjacent to the population of skeletal precursor cells, suppresses joint formation [7]. For example, the *sof* ^*b123*^ mutant (reduced *cx43*) exhibits short segments/premature joints. Therefore, we predicted that the expression of the joint genes would initiate sooner, or more distally, in *sof* ^*b123*^ fins than in wild type. Indeed, expression of *evx1*, *dlx5a*, and *mmp9* genes are each initiated more distally in *sof* ^*b123*^ regenerating fins compared with wild type (Table 1). These findings are consistent with the reduced level of Cx43 activity causing premature activation of the *evx1*-dependent joint pathway, and premature joints. It may be suggested that the reduced growth rate of *sof* ^*b123*^ causes the shift of gene-expression domains to more distal locations. However, such a shift in patterning due to differential growth rates has not been observed. For example, fin amputations at more proximal locations regenerate more rapidly than fin amputations at more distal locations. However, when comparing these conditions for four different genes located in the basal layer of the epidermis (i.e. *lef1*, *shh*, *wnt5b*, *pea3*), the distance of expression of the gene domain to the distal end did not appear altered, although the strength of expression and/or size of the expression domain can change [21]. Therefore, to confirm that the reduced growth rate of *sof* ^*b123*^ does not influence the patterning of gene expression in general, we compared the expression of both *shh* and *lef1* between *sof* ^*b123*^ and wild-type regenerating fins. Importantly, we found no significant changes in the distance of the distal expression domains for either gene to the distal end of the fin between wild type and *sof* ^*b123*^ (although we do see that overall expression levels are slightly reduced) (Figure 7). Thus, the reduced growth rate of *sof* ^*b123*^ is likely not the cause of the distal shift in joint gene expression. Rather, we suggest that reduced *cx43* in *sof* ^*b123*^ leads to premature expression of the joint genes.

**Table 1:** Expression domains of genes contributing to joint formation in the regenerating fin.

	WT	*sof ^b123^*	*alf ^dty86^*
*evx1*	(+) 75.47 μm ± 6.2 μm N=9	(+) 64.1 μm ± 4.36 μm N=9	(+/-) 109.33 μm ± 20.7 μm N=9
*dlx5a*	(+) 106.80 μm ± 15.21 μm N=8	(+) 79.16 μm ± 10.26 μm N=8	(+) 110.52 μm ± 22.15 μm N=15
*mmp9*	(+) 128.98 μm ± 16.11 μm N=8	(+) 86.34 μm ± 14.65 μm N=8	(+) 110.73 μm ± 19.55 μm N=15

The distance is measured from the expression domain (determined by whole mount ISH) to the distal end of the fin, using the third fin ray as a standard, as previously established [19]. Irregular (+/-) expression of *evx1* was observed in the *alf* ^*dty86*^ fins, while *dlx5a* and *mmp9* gene expression was present in all fin rays (+). The *evx1* expression domain in the *alf* ^*dty86*^ fins was measured from the subset of *evx1*-positive fin rays. N, number of fins.

**Figure 8:**
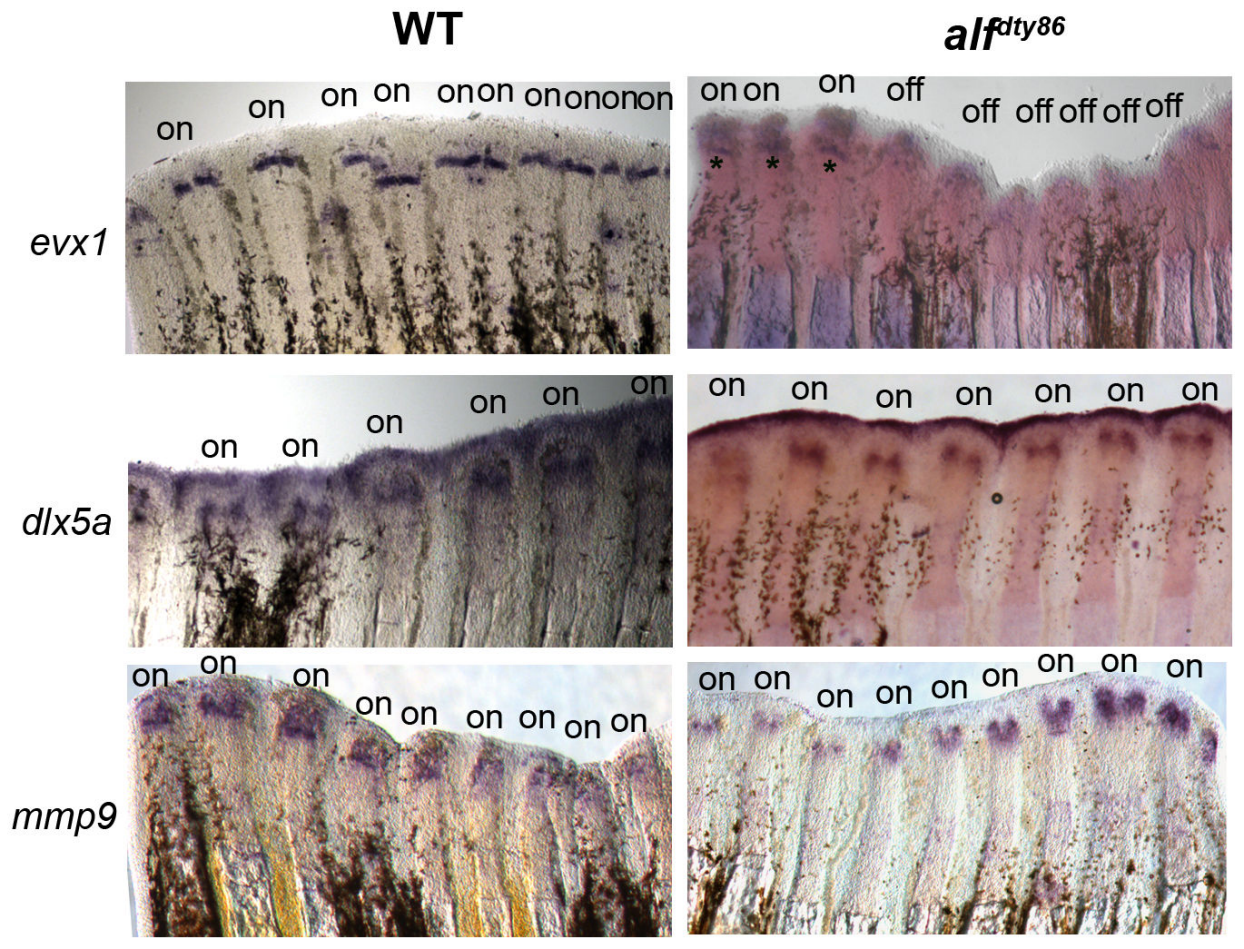
Expression of joint genes in *alf* ^*dty86*^. Whole mount ISH of *evx1* shows that *evx1* is expressed consistently in wild-type but irregularly in *alf* ^*dty86*^ (i.e. “on” vs. “off”) Astericks were placed just proximal to the *evx1*-positive staining in *alf* ^*dty86*^, which are present even though expression is weak. In contrast, *dlx5a* and *mmp9* are expressed in all fin rays in both wild-type and *alf* ^*dty86*^ fins.

**Figure 9:**
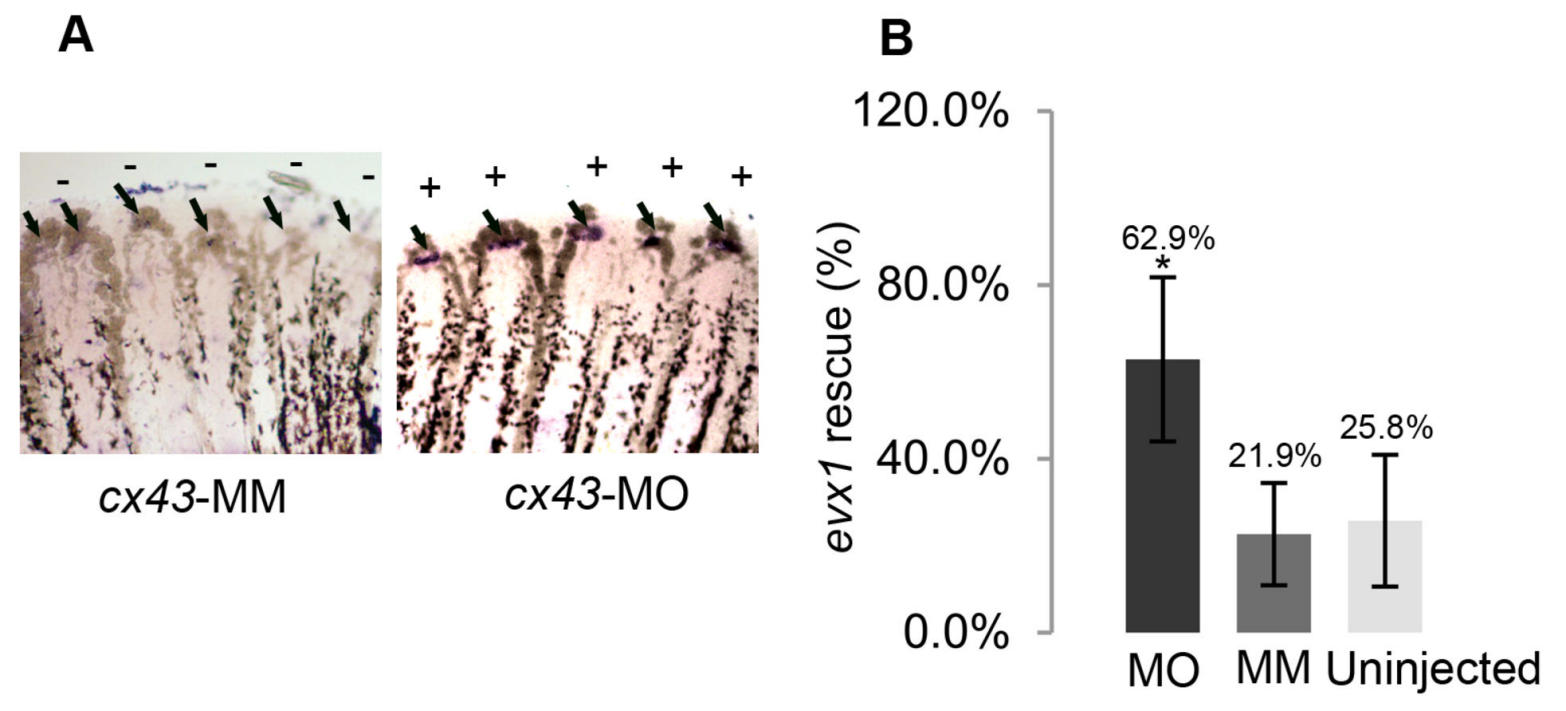
Knockdown of *cx43* rescues *evx1* expression in *alf* ^*dty86*^. (A) Whole mount ISH of *evx1* shows that *evx1* is expressed in most fin rays in *cx43*-morpholino (MO) fins (on) but not in *cx43*-mismatch (MM) fins (off). Arrows identify *evx1*-positive signal. (B) *evx1* expression is present in a much higher percentage of fin rays in *alf* ^*dty86*^ following injection/electroporation of *cx43*-MO compared with either the *cx43*-MM control fins or the uninjected fin rays. Statistical significance (*) was determined by student’s t-test where p<0.05 show significant differences. The p-value for the comparison of *cx43*-MO and *cx43*-MM was p = 0.0015. The p-value for the comparison of *cx43*-MO and uninjected fin rays was p = 0.0036. The bars represent the standard deviation.

We next examined joint gene expression in the *alf* ^*dty86*^ mutants, which exhibit stochastic joint failure and overlong segments on average due to increased expression levels of *cx43* [7,18]. Thus, in *alf* ^*dty86*^ we expected to observe an irregular pattern (on/off) of joint gene expression and/or more proximal expression of the joint genes compared with wild type. Indeed, *evx1* is expressed in a stochastic pattern and also initiates more proximally (Figure 8 and Table 1). Since *evx1* is required for joint formation [15], these findings suggest that the stochastic nature of *evx1* expression is the underlying cause of stochastic joint failure in *alf* ^*dty86*^. Moreover, we suggest that the increased level of *cx43* in *alf* ^*dty86*^ is the underlying cause of stochastic *evx1* expression (i.e. since *cx43*-knockdown rescues joint formation in *alf* ^*dty86*^, [7]). Therefore, we next wished to determine if *cx43*-knockdown rescues *evx1* expression. We tested this by injecting either a *cx43*-targeting morpholino or a *cx43*-mismatch morpholino across all fin rays in *alf* ^*dty86*^ regenerating fins. Next, the percentage of *evx1*-positive fin rays was determined for each fin. We find that *cx43*-knockdown in *alf* ^*dty86*^ regenerating fins significantly increases the percentage of *evx1*-positive fin rays compared with the *cx43*-mismatch morpholino and compared with uninjected *alf* ^dty86^ regenerating fins (Figure 9). These findings reveal that *cx43*-knockdown relieves the suppression of *evx1* expression, thereby permitting joint formation. Therefore, we suggest that *cx43* suppresses joint formation by suppressing *evx1* expression.

It was anticipated that the stochastic nature of *evx1* expression would lead to stochastic expression of both *dlx5a* and *mmp9*. However, this was not observed (Figure 8). Instead, we find their expression is activated in all fin rays in *alf* ^*dty86*^, consistent with the observation that *dlx5a* and *mmp9* expression are not completely eliminated in the *evx1* ^*-/-*^ mutant, and therefore appear to be activated even in the absence of *evx1*. Since joint failure occurs despite expression of *dlx5a* and *mmp9*, these data also suggest that *dlx5a* and *mmp9* cannot mediate joint formation without the additional expression of *evx1*. Thus, since *evx1* is required for joint formation but *dlx5a* and *mmp9* are required but not sufficient for joint formation, *evx1* must activate at least one other pathway to establish fin ray joints. Continued studies are required to identify this pathway.

### Model of joint differentiation during fin regeneration

**Figure 10:**
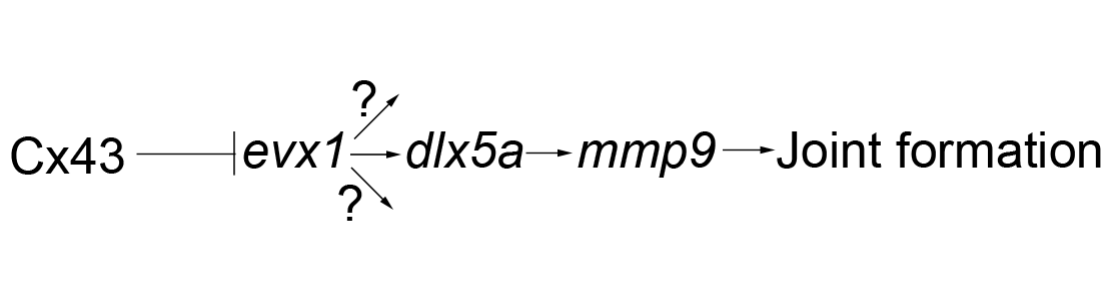
Model of the identified joint pathway. Both *dlx5a* and *mmp9* appear to be regulated by both *evx1*-dependent and non-*evx1*-dependent manners. In addition, *evx1* may activate additional genes required for joint formation. Cx43 appears to regulate joint differentiation by influencing *evx1* expression.

Our analyses of joint gene expression suggest a model for joint formation that requires *evx1*, which is expressed the earliest, followed by expression of *dlx5a* and *mmp9* (Figure 10). Each of these genes is expressed in the population of skeletal precursor cells, adjacent to the Cx43-positive medial mesenchyme, and all three genes contribute to joint formation. We further suggest that Cx43 influences joint formation by influencing *evx1* expression. When *cx43* activity is reduced, as in *sof* ^*b123*^, expression of all *evx1*-dependent joint genes is shifted distally, consistent with the observation that *sof* ^*b123*^ produces premature joints. When *cx43* activity is increased, as in *alf* ^*dty86*^, expression of *evx1* is irregular, consistent with the stochastic joint failure observed in *alf* ^*dty86*^ regenerating fins. Indeed, *cx43*-knockdown rescues both *evx1* expression and joint formation [7] in *alf* ^*dty86*^. Interestingly, expression of *dlx5a* and *mmp9* are not randomized, but instead are consistently expressed in all fin rays. Continued studies will be necessary to identify additional possible *evx1*-dependent pathways, and to understand how *dlx5a* and *mmp9* expression is maintained in the absence of *evx1*.
